# Raising the Political Profile of the Neglected Zoonotic Diseases: Three Complementary European Commission-Funded Projects to Streamline Research, Build Capacity and Advocate for Control

**DOI:** 10.1371/journal.pntd.0003505

**Published:** 2015-03-05

**Authors:** Anna L. Okello, Iona Beange, Alexandra Shaw, Ignacio Moriyón, Sarah Gabriël, Kevin Bardosh, ICONZ, Maria Vang Johansen, Christopher Saarnak, Samson Mukaratirwa, ADVANZ, Dirk Berkvens, OH-NEXTGEN, Susan C. Welburn

**Affiliations:** 1 Division of Pathway Medicine and Centre for Infectious Diseases, School of Biomedical Sciences, College of Medicine and Veterinary Medicine, The University of Edinburgh, Edinburgh, United Kingdom; 2 Agriculture and Veterinary Information and Analysis (Avia-GIS), Zoersel, Belgium; 3 Instituto de Salud Tropical y Depto Microbiología y Parasitología, Universidad de Navarra, Pamplona, Spain; 4 Institute of Tropical Medicine, Antwerp, Belgium; 5 Centre of African Studies, School of Social and Political Science, The University of Edinburgh, Edinburgh, United Kingdom; 6 Section for Parasitology and Aquatic Diseases, Department of Veterinary Disease Biology, Faculty of Health and Medical Sciences, University of Copenhagen, Copenhagen, Denmark; 7 School of Life Sciences, University of KwaZulu‐Natal, Durban, South Africa; University of Liverpool, UNITED KINGDOM

## Introduction

The World Health Organisation identifies eight Neglected Zoonotic Diseases (NZDs) as major causes of ill health to both humans and domestic animals in many countries across the world [1–3]. Although largely controlled or eradicated in industrialised nations, these eight NZDs—anthrax, brucellosis, bovine tuberculosis (bTB), *Taenia solium* cysticercosis, cystic echinococcus, leishmaniasis, rabies, and human African trypanosomiasis (HAT)—still cause significant health problems in many low resource settings in developing nations [1–7]. The poor remain disproportionately affected by NZDs through a combination of close contact with domestic animals (particularly in rural settings), and the difficulties of accessibility, affordability, and capacity of local health centres. Aside from the significant human mortality and morbidity caused by NZDs, their added impact on livestock productivity—including decreased fecundity, poor growth rates, lowered draft power outputs, decreased carcass value, and deadly epidemics—further contribute to the burden of NZDs on poor livelihoods. NZD control offers a powerful opportunity to simultaneously save lives and secure livelihoods, contributing to poverty alleviation within affected communities [2].

History provides compelling evidence that the effect of zoonoses in humans can be mitigated through targeted interventions in animal reservoirs; the successful eradication of brucellosis, porcine cysticercosis, bTB, and rabies from a number of countries has all been done this way [8–10]. However, given the significant benefits of NZD control and prevention to the broader human health and development sectors, the financial and logistical responsibility for zoonoses control should not just fall on the shoulders of the veterinary sector alone. Confusion over roles and responsibilities, often resulting from the perception that the NZDs are beyond the mandate of either the human or animal health sectors, currently impede concerted efforts towards their control [11]. Vast underreporting, often as a result of poor advocacy, diagnostic difficulties and disease clustering that may be missed in broad-based epidemiological surveys, further compounds their political “neglect.” At the local level, primary healthcare workers and local veterinary officers faced with poor infrastructural investments in both health and veterinary services, lack the information, knowledge, and tools for NZD diagnosis and control. Moreover, the societal value of livestock and lack of compensation programmes render standard control methods utilised in industrialised nations, such as test and slaughter, unimplementable and unacceptable in much of Africa, Asia, and Latin America [12].

Despite the multiple benefits of control, coordinated efforts to collectively address the NZDs is generally lacking. With recent estimations that broader Neglected Tropical Disease (NTD) funding represents just 0.6% of total international development assistance [13], funding for the NZDs has been estimated at around one-tenth of this figure; a mere 0.06% of global assistance for health [3]. The need to identify and quantify the impact of endemic zoonoses in developing regions, evaluate and prioritise control approaches, and build national and local capacity and leadership is imperative. The European Commission, through their Seventh Framework Programme (FP7), has funded three complementary projects to address these issues on a large scale in Africa; i) Integrated Control of Neglected Zoonoses in Africa (ICONZ), ii) training of the One Health Next Scientific Generation in the Sahel and Maghreb (OH-NEXTGEN), and iii) Advocacy for Neglected Zoonotic Diseases (ADVANZ). Through simultaneously generating evidence, building capacity, and advocating for control, these three programmes promote coordination and collaboration for increasing the political visibility of this important, but underfunded, group of diseases (Table 1). The remainder of this article highlights the objectives and activities of these three projects, and discusses the policy implications for NZD control expected to arise from their outputs.

**Table 1:** Major deliverable thematic areas highlighting complementary areas.

**Deliverable**	**ICONZ**	**ADVANZ**	**OH-NEXTGEN**
Project governance, coordination, and reporting	WP1	WP5	WP7
Interactive project website	WP12	WP1	WP3
Policymakers appraised of importance of NZDs, including burdens and cost in humans and animals	WP5–9, WP12	WP1, WP3–4	WP4–5
Virtual training and advocacy platforms and networks	WP11	WP1, WP3	WP3, WP5
Scientific publication in form of individual research papers and dedicated journal issues	ALL WPS	ALL WPS	ALLWPS
Institutional and organisational linkages	WP9, WP12	WP1	WP4
Advocacy material and awareness raising	WP3, WP5–10, WP12	WP1–3	WP5
Gap analysis of NZD diagnosis, burdens, controls, institutional, training, and messaging aspects	WP2, WP4, WP9–11	WP1–3	WP2
NZD fourth international meeting		WP4	
Learning materials and study guides for NZDs	WP11	WP3	WP1–2
Course evaluation and certification	WP11		WP4
Project newsletters	WP12		WP7
Standardised methodology for quantifying NZD societal burdens and costs of control	WP3, WP9		
Disease surveillance and transmission data including spatial analysis	WP3		
Improved NZD control and prevention strategies	WP3, WP5–9		
Integrated cost-effective disease control packages	WP3, WP5–9		
Comparative analysis of control costs, methods, and recommendations	WP9		
Sociocultural aspects of NZD transmission and control	WP10		
National country reports on disease factors and policy implications	WP12		

## ICONZ (www.iconzafrica.org)

### Concept and Objectives

The ICONZ project has been specifically designed to generate an evidence base for the promotion of integrated control packages for the eight NZDs in seven African International Partner Cooperation Countries (ICPCs): Morocco, Mali, Nigeria, Uganda, Tanzania, Mozambique, and Zambia. Integrated control refers to interprogrammatic and intersectoral approaches based on stratification of risk to reach marginalized populations or geographic areas, rather than promoting vertical strategies that address each disease independently [14,15]. Scientific innovation and public engagement remain two important cornerstones to the ICONZ approach; locally-appropriate strategies are mindful of the wider existing policy frameworks of affected countries, with sustainability of these approaches ensured through the training of almost 70 postgraduate students (Masters and PhD) from both Africa and Europe to date. Whilst African countries have been the focus of ICONZ, given that it is the only continent affected by all eight zoonoses targeted by the FP7 call, it is anticipated that the strategies and experience resulting from ICONZ research can help form recommendations and advise other countries suffering similar burdens of disease, particularly in Asia and Latin America.

**Fig 1:**
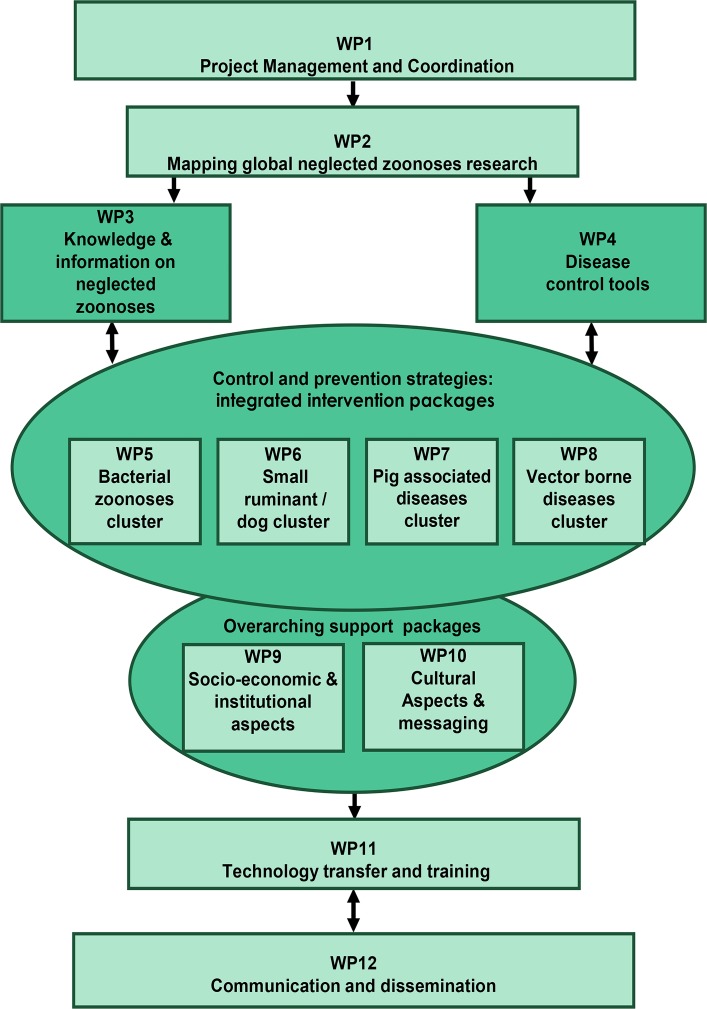
Overview of ICONZ WPs.

The overall strategic objective of ICONZ is to mitigate the human and animal health impacts of NZDs, whilst contributing to poverty alleviation and the Millennium Development Goals. In order to achieve this, twelve Work Packages (WPs) incorporating 21 European and African partners have joined forces to determine the current disease burdens on communities, and subsequently identify innovative approaches for NZD identification and control tailored to different settings (Fig. 1). The ICONZ approach has emphasised the need to communicate research evidence to the highest possible number of beneficiaries and decision makers within each ICPC, to maximise impact on the local and national scale.

### Overview of ICONZ WPs

#### ICONZ WPs 1–4: Project management and gap analyses

These four WPs align to ensure the effective management of interactions between the project partners, donors, and beneficiaries (WP1), create a database of concurrent NZD research in other parts of the world (WP2), cost and standardise methodologies for evaluating disease burden (WP3), and validate currently available diagnostic tools (WP4). Through fulfilling the deliverables of each of these WPs, it is anticipated that a clear analysis of the evidence gaps, and appropriate methodologies to address these, will be available to guide the activities of the remaining eight WPs.

#### ICONZ WPs 5–8: Development of integrated intervention packages for NZD clusters

The backbone of ICONZ consists of four WPs focusing on the development of integrated “packages” of interventions, targeted at groups or “clusters” of related NZDs. Each WP represents a disease cluster, stratified according to similarities in their hosts, transmission, or control factors. WP5 (bacterial zoonoses) has a focus on anthrax, bTB, and brucellosis in Morocco, Nigeria, and Tanzania. Whilst there currently exists no animal intervention for bTB outside a test and slaughter policy, conjunctival brucellosis vaccines have been trialled in cattle in Nigeria, and community-based education interventions around common risk factors for both brucellosis and bTB have been undertaken in Tanzania. Moreover, data obtained in each of these countries provides insights on the prevalence under different management conditions that should be valuable to propose locally adapted control strategies. WP6 (dog and small ruminant zoonoses) is responsible for rabies, leishmaniasis, and cystic echinococcus in Morocco and Mali. In an innovative dog intervention in Morocco, researchers vaccinate, deworm, and place an insecticide-treated collar on dogs in the project areas, simultaneously targeting rabies, echinococcus (and other canine internal parasites), and the sand fly vector that causes leishmaniasis. The main focus of WP7 (pig-associated parasitic zoonoses) is *T*. *solium*, where assessment of community-based interventions such as Community-led Total Sanitation (CLTS), health education, and pig management has occurred in Zambia and Mozambique, respectively. WP8 (vector-borne zoonoses) addresses HAT in Uganda through rolling out a series of Restricted Application Protocol (RAP) regimes for insecticidal treatment of cattle against the tsetse fly vector, assessing the various economic and epidemiological factors associated with each regime. The overall objectives for these four WPs is to develop and test integrated, cost-effective control strategies for each NZD cluster, considering various context-specific economic, social, and cultural aspects. Additionally, WPs 5–8 provide technical information to guide training, policy, and advocacy activities mandated to WPs 9–12.

#### ICONZ WPs 9–12: Support and dissemination

Understanding local sociocultural contexts, policy processes, and economic factors are essential to drive the integrated and cross-sectoral control strategies required for NZD control. Messaging needs to be tailored to a large variety of stakeholders and beneficiaries to promote a long-term commitment. WP9 (socioeconomic and institutional aspects) and WP10 (sociocultural and messaging) address these crosscutting issues, providing overarching support to the design of control strategies outlined in WPs 5–8. WP11 (technology transfer) and WP12 (communication) are ultimately responsible for transferring the knowledge gained from ICONZ to the wider scientific, political, and community-level spheres, in conjunction with the two other European Union (EU)-funded NZD projects discussed below, OH-NEXTGEN and ADVANZ.

## OH-NEXTGEN (www.oh-nextgen.eu)

### Concept and Objectives

Despite the rhetoric around the added benefit of applying a One Health approach to national disease surveillance and control, ongoing institutional barriers largely limit the opportunities for closer cooperation between the human and animal health sectors [5,16]. Notwithstanding the impressive developments in One Health at both the national and international level in recent years [17], a large gap still remains. In many ways, such collaborative failures are embedded within accepted norms of professionalism established during training, particularly in prevailing teaching approaches that tend to compartmentalize disciplines, leading to downstream collaboration failures. OH-NEXTGEN is tasked with the development of a web-based modular training course designed to empower a new generation of scientists to transcend silos in order to address One Health issues faced by communities in Africa.

The specific objectives of the project include the development, delivery, and evaluation of a One Health postgraduate curriculum, training of local tutors from a wide range of medical, veterinary, environmental, and biomedical backgrounds, and production of a web platform that promotes an interactive virtual learning environment. While the programme is currently targeted to the Maghreb and the Sahel, the course will ultimately be accessible worldwide by offering training modules through the European Tropical Health Education Network (tropED) and other existing platforms. The course combines specific technical information regarding the NZDs with overarching themes such as integrated surveillance platforms, health economics, policy process analysis, and transdisciplinary approaches for addressing cultural and gender issues, demonstrating the added value of One Health approaches within each module.

### Overview of WPs

**Fig 2:**
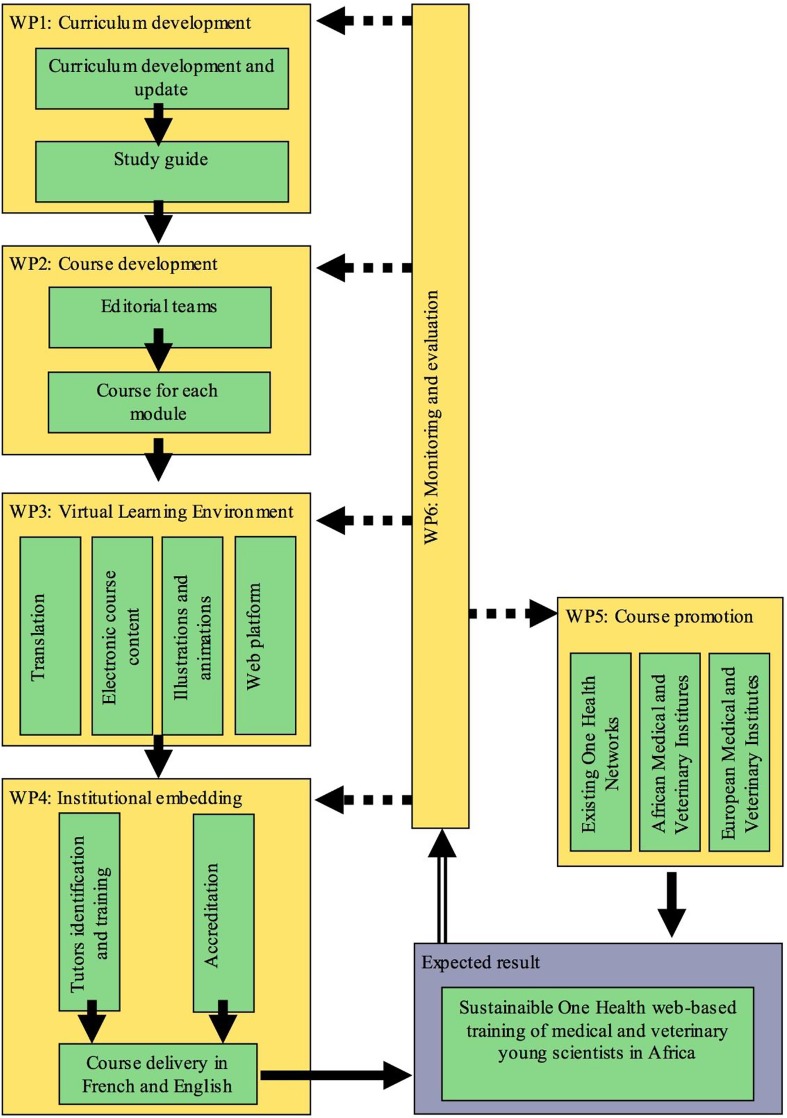
Overview of OH-NEXTGEN WPs.

Seven WPs are responsible for the development and delivery of the online training programme, in addition to quality control and other tools for evaluation (Fig. 2).

#### OH-NEXTGEN WPs 1–4: Curriculum development, delivery, and integration into existing institutions

Nine teaching modules—equating to 30 credits (postgraduate certificate level)—have been developed, offering both basic and advanced modules of the following topics: One Health, epidemiology, socioeconomics, policy development, health education, and communication. One Health constitutes a strong foundation across each module, combining validated and theoretical examples to demonstrate the added institutional and socioeconomic benefits of an integrated approach to NZD control. The French language “training of trainers” face-to-face teaching course occurred in February 2014 in Rabat, Morocco, with the English language course scheduled for early 2015 in Accra, Ghana.

#### OH-NEXTGEN WPs 5–8: Management, communication, quality, and sustainability

It is envisioned that the OH-NEXTGEN course materials will become available to candidates across Africa, using appropriate formats such as policy briefs, newsletters, and web-based information services to also encourage participation from nonacademic stakeholders. In this way, quality assurance will ensure the course content remains aligned with the needs of this wide audience.

## ADVANZ (www.advanz.org/)

### Concept and Objectives

ADVANZ aims to empower stakeholders at the local, regional, and international levels to target the NZD burdens in Africa. A major challenge for control is that symptoms of NZDs can remain silent for a long time, or be easily confused with other illnesses, making it difficult to advocate for behaviour change of infected communities, or implement control policies at the national level. The development and dissemination of locally specific, easily accessible information is fundamental to generate awareness and alter risk-associated practices and behaviours.

ADVANZ targets national decision makers involved in disease control at three key levels: the ministerial level (primarily stakeholders at the ministries of health and agriculture), the district level (health and veterinary officers or extension workers who require further information on the NZD impact in their communities), and the local communities themselves. Innovative, locally relevant, and context specific communication tools have been developed, incorporating evidence generated by the ICONZ case studies. Examples include policy briefs navigating the complex political sphere of global health and smart phone applications to facilitate information flow on NZDs and risky behaviours.

### Overview of WPs

#### ADVANZ WPs 1–3: Pan‐African NZD platform and advocacy database

**Fig 3:**
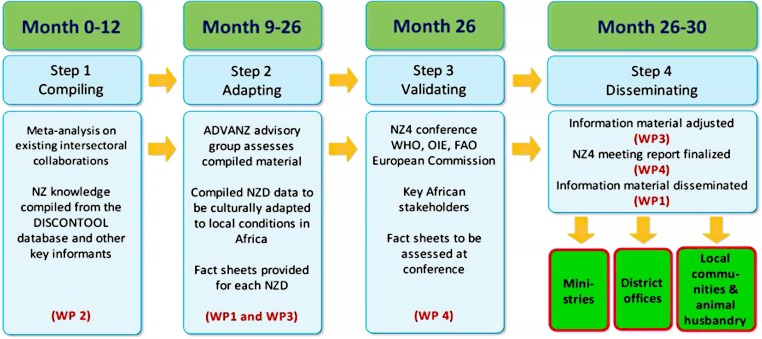
Overview of ADVANZ WPs.

Fig. 3 details the objectives of the four ADVANZ WPs. The main objective of WP1 is to maintain the recently established Pan‐African platform for advocacy and knowledge dissemination on the NZDs (http://www.advanz.org/pan-african-one-health-platform/). The platform acts as a link among the existing NZD networks and institutions in Africa, compiling and providing information and disease specific material for various stakeholders involved in the control and prevention of NZDs. The information material has been compiled from the vast existing body of knowledge currently available. Particular attention is paid to evidence of successful and profitable intersectoral collaboration in low resource societies. The information is supported by the most recent findings from the ICONZ research, forming WP2. ADVANZ WP3 has created a digital platform for disseminating the knowledge in the form of a website under the Pan-African platform website, as well as stand-alone smart phone applications targeted to the African end user.

#### ADVANZ WP 4: Fourth international meeting on NZDs—“From Advocacy to Action” (http://www.who.int/neglected_diseases/zoonoses/fourth_international_nzd_meeting/en/)

The final outcome of ADVANZ was the fourth in a series of international NZD stakeholder meetings that commenced in 2005. NZD4, held in November 2014 and hosted by the World Health Organisation in Geneva, was a forum for exchange between decision makers, health researchers, and donors across the international, regional, and national domains. A key focus throughout this fourth meeting was how to translate the available research evidence into appropriate policy frameworks for NZD control. The resulting report, to be printed in early 2015, will complement the current WHO series [1–3].

## Conclusion: Policy Significance of the Three Research Projects

Through supporting these three ambitious NZD programmes within its FP7 Programme, the European Commission is leading the fight against this group of important but neglected diseases, simultaneously promoting livestock productivity, human health, and overall development in high-risk, low-resource societies across Africa. Additionally, all three projects encompass a One Health focus, where the positive benefits of collaborative approaches in poor-resource settings are gaining attention [5,18]. The high quality epidemiological, socioeconomic, and sociocultural data generated by ICONZ will result in a greater understanding of NZD burden, transmission, and control, essential for garnering political support for future research and control programmes amongst the international community. ICONZ outputs will also help define priority policy areas for national research and advocacy in Africa, further promoted and supported by building a new generation of African One Health scientists and decision makers trained through OH‐NEXTGEN based on capacity. Overarching the research and capacity building activities, ADVANZ will foster the ongoing transfer of NZD knowledge from the grass roots to the highest political levels, advocating through a variety of media platforms for sustainable NZD control. Whilst these three projects undeniably highlight the growing global movement towards One Health approaches to tackle the NZDs, the expansion of NZD-focused research and policy action throughout Africa, and into other regions, will ultimately depend on sustaining and expanding the positive momentum gained thus far through ICONZ, OH-NEXTGEN, and ADVANZ.
